# Functional Characterization of the Arabidopsis Abscisic Acid Transporters NPF4.5 and NPF4.6 in Xenopus Oocytes

**DOI:** 10.3389/fpls.2020.00144

**Published:** 2020-02-26

**Authors:** Sophie Léran, Mélanie Noguero, Claire Corratgé-Faillie, Yann Boursiac, Chantal Brachet, Benoit Lacombe

**Affiliations:** BPMP, Univ Montpellier, CNRS, INRAE, Montpellier SupAgro, Montpellier, France

**Keywords:** abscisic acid, transport, hormone, Km, pH

## Abstract

Few proteins have been characterized as abscisic acid transporters. Several of them are NRT1/PRT Family (NPF) transporters which have been characterized in yeast using reporter systems. Because several members of the NPF4 subfamily members were identified in yeast as ABA transporters, here, we screened for ABA transport activity the seven members of the NPF4 subfamily in Xenopus oocytes using cRNA injection and ^3^H-ABA accumulation. The ABA transport capacities of NPF4.2, NPF4.5, NPF4.6, and NPF4.7 were confirmed. The transport properties of NPF4.5 and NPF4.6 were studied in more detail. Both ABA transporter activities are pH-dependent and slightly pH-dependent apparent Km around 500 μM. There is no competitive inhibition of the ABA-analogs pyrabactin and quinabactin on ABA accumulation demonstrating a different selectivity compared to the ABA receptors. Functional expression of these ABA transporters in Xenopus oocyte is an opportunity to start structure–function studies and also to identify partner proteins of these hormone transporters.

## Introduction

The weak acid sesquiterpene abscisic acid (ABA) was identified in plant in the sixties (Zhang, 2014). It is widely described as the stress hormone because it is involved in the plant responses to many biotic and abiotic environmental signals (Cutler et al., 2010). From its discovery to 2010, most of the work was dedicated to the identification of biosynthetic and catabolic pathways, and several enzymes involved in these processes have been identified (Nambara and Marion-Poll, 2005). In 2009, the perception and signaling pathways came back to light with the identification of the ABA receptors from the PYR/PYL/RCAR family (Ma et al., 2009; Park et al., 2009).

While long-distance ABA transport within the plant was characterized years ago, the firsts ABA transmembrane transporters were only identified in 2010 (Boursiac et al., 2013). What are the ABA transporters identified so far?

The first family of protein which have ABA transporters is the ABCG subfamily of ABC (ATP BINDING CASSETTE) transporters, with four of them related to ABA transport: ABCG25, 30, 31, and 40. ABCG25 exports ABA from the vascular parenchyma cells and AtABCG40/PDR12 mediates guard cells ABA uptake to trigger stomatal closure (Kang et al., 2010; Kuromori et al., 2010; Kuromori and Shinozaki, 2010; Kuromori et al., 2016; Kuromori et al., 2018). These transporters, together with AtABCG30 and AtABCG31, are involved in seed dormancy. AtABCG25 and AtABCG31 export ABA from the endosperm whereas AtABCG30 and AtABCG40 import ABA in the embryo to suppress seed germination (Kang et al., 2015). The Medicago MtABCG20 is an ABA exporter involved in root development and seed germination (Pawela et al., 2019).

The second family of ABA transporters is the NPF (NRT1/PTR FAMILY) (Corratgé-Faillie and Lacombe, 2017). An elegant functional screen of ABA transport in yeast was used to identify NPF4.6/AIT1/NRT1.2, NPF4.5/AIT2, NPF4.1/AIT3, and NPF4.2/AIT4 (Kanno et al., 2012). Their ABA transporting activities were tested and confirmed by ABA accumulation studies in either yeast cells or *Sf*9 cells (Kanno et al., 2012). NPF4.6 is expressed around vascular tissues, and mutants defective in AtNPF4.6 have lower surface temperatures than the wild-type, supporting a role as an ABA transporter *in planta* (Kanno et al., 2012). NPF4.6 is also a nitrate transporter (Huang et al., 1999), so the effect of nitrate on ABA accumulation has been tested (Kanno et al., 2013), but an interaction between the two substrates has not been demonstrated. Using the same ABA-dependent two-hybrid system and screening 45 out of the 53 Arabidopsis NPF members, Chiba and coworkers (Chiba et al., 2015) confirmed that NPF4.6, NPF4.1, and NPF4.5—and also additional NPF members such as NPF1.1, NPF2.5, NPF5.1, NPF5.2, NPF5.3, NPF5.7, and NPF8.2—are ABA influx transporters. More recently, Tal et al. (2016) have demonstrated the ability of NPF3.1-expressing oocytes to accumulate ABA. The Medicago MtNPF6.8 is an ABA influx transporter when expressed in Xenopus oocytes (Pellizzaro et al., 2014).

Two other proteins behave as ABA transporters. A DTX/MATE (Detoxification efflux carrier/multidrug and toxic compound extrusion), AtDTX50 is an Arabidopsis efflux transporter involved in ABA sensitivity and drought tolerance (Zhang et al., 2014). In rice, an AWPM-19-family member (OsPM1, PLASMA MEMBRANE PROTEIN1) is an ABA influx transporter involved in drought response (Yao et al., 2018).

Despite the number and the diversity of the ABA transporters, the detailed transport properties of these proteins are largely unknown. The aims of our work were: (i) to identify functional ABA transporters within the 7 NPF4 proteins, using heterologous expression and ^3^H-ABA and (ii) to perform a detailed characterization of the functional properties of NPF4.5 and NPF4.6. Besides its numerous advantages for membrane transport characterization, the use of Xenopus oocytes also gives the opportunity to determine the transport parameters in other systems.

## Materials and Methods

### Plasmids and cRNA Synthesis

NPF coding sequences (CDS) were either obtained from ABRC (cloned in pENTR223 for NPF4.3, 4.5) or cloned in pENTR/D/TOPO (for clones NPF4.1, 4.2, 4.4, 4.7), and pDONR207 (for clones NPF4.1, 4.6). Each clone was sequenced and compared to Col-0 genomic sequence. LR reaction was performed according to the manufacturer's instructions (Life Technologies), to clone the CDS into the Xenopus oocyte expression vector [pGEM-GWC, (Leran et al., 2015)].

### Oocytes Expression

NPFx-pGEM-GWC vectors were linearized and *in vitro* transcribed with mMessage mMachine T7 Ultra Kit following manufacturer protocol (Life Technologies). Xenopus oocytes were purchased from the Centre de Recherche en Biochimie Macromoléculaire (CNRS, Montpellier, France). Oocytes were obtained and injected as previously described (Lacombe and Thibaud, 1998).

### ABA Uptake Experiments and ^3^H-ABA Quantification

For ABA uptake, oocytes were incubated for 20 min in 1 ml of ND96 solution (pH indicated in the figure legends) containing the indicated concentration of ABA (10% of the labeled ^3^H-ABA, American Radiolabelled Chemicals and 90% of cold ABA, Sigma). They were then washed 4 times in 15 ml of ND96 solution (4°C) containing 5 µM of cold-ABA. Each oocyte was then dissolved in 100 µl of 2% Sodium Dodecyl Sulfate (SDS). Lysis solution was then mixed to 3 ml of scintillating solution (ULTIMAGOLD, PerkinElmer). Incorporated radioactivity was measured by Liquid-Scintillation analyzer (Tri-Carb 2100 TR, Perkin Elmer).

### Fitting Procedure

Least squares fit using SIGMAPLOT (11.0, Systat Software Inc.) has been used. The ABA concentration range was between 0 and 5 μM ^3^H-ABA. Data were fitted by a Michaelis–Menten equation: A = (Amax * [ABA])/(Km + [ABA]), where A is the intracellular ABA accumulation, Amax is the maximum intracellular accumulation, (ABA) is the external ABA concentration and Km is the apparent affinity.

## Results

### Expression of the Seven AtNPF4 in Xenopus Oocytes

**Figure 1:**
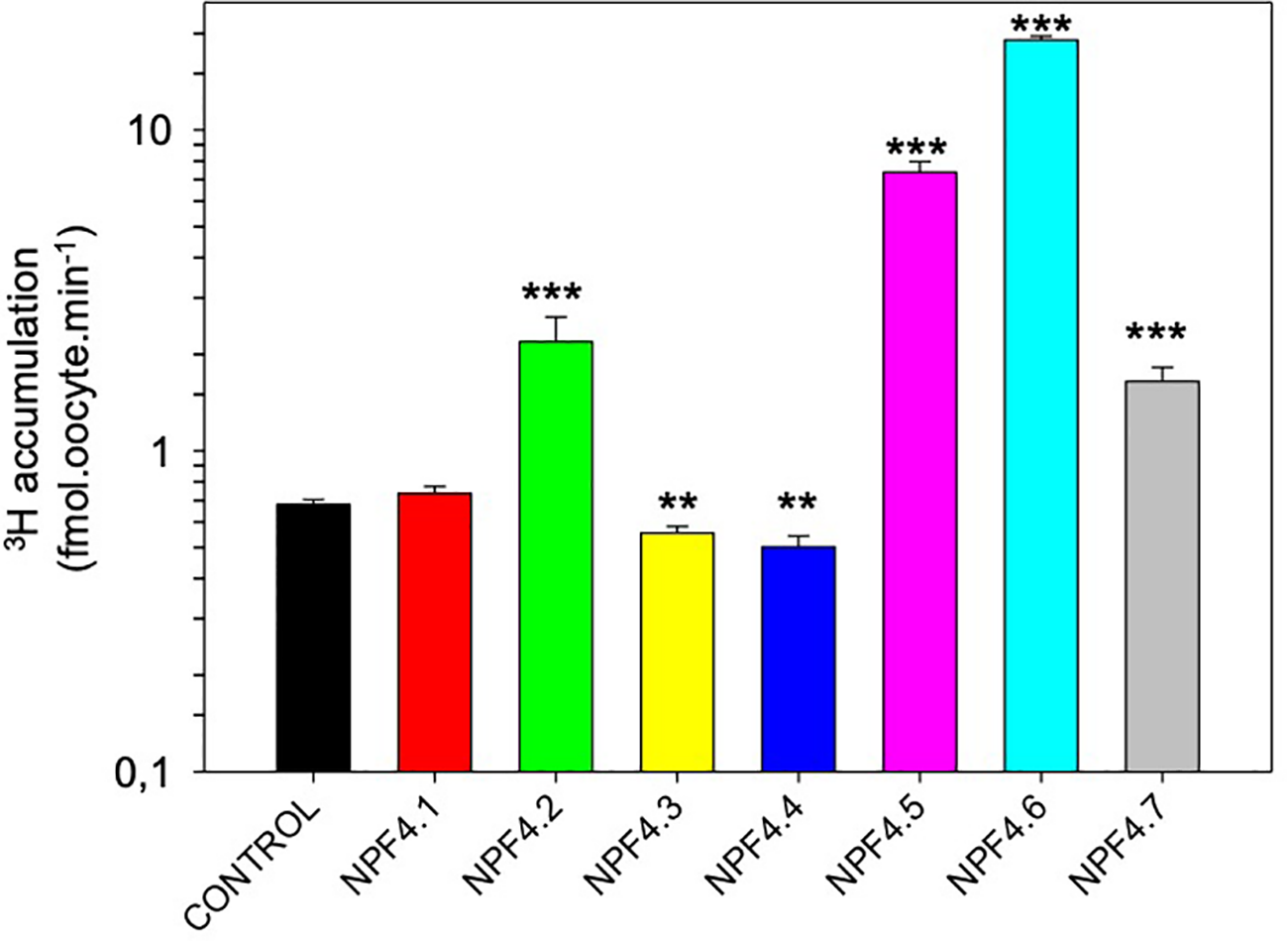
Screen for ABA transport activity of Arabidopsis NPF4 subfamily members in Xenopus oocytes. Control (noninjected) and NPF4-injected oocytes were bathed in 1 μM ^3^H-ABA (pH = 6.0), and ^3^H accumulation in oocytes was quantified after 20 min. Values are mean +/− SEM (n = 5–12 oocytes, biological replicates). ***P < 0.001, **0.001 < P < 0.005, two-sided t-test after comparison with control oocytes.

Xenopus oocytes are used to express the seven Arabidopsis NPF4 proteins after injection of *in vitro* transcribed cRNA. Noninjected oocytes were used as negative controls. We used ^3^H-labeled ABA as a tracer for ABA accumulation into oocytes. After 20 min incubation in ^3^H-ABA containing ND96 solutions, ^3^H was quantified into oocytes (**Figure 1**). Control oocytes accumulate low levels of ^3^H, this could be explained by the membrane diffusion of protonated form of ABA (ABA-H). Whereas in yeast NPF4.1 is an ABA influx transporter (Kanno et al., 2012; Kanno et al., 2013; Chiba et al., 2015), NPF4.1-expressing oocytes accumulate ^3^H at the same level as the control. NPF4.2 and NPF4.7-expressing oocytes accumulate more than 2.5-fold ^3^H compared to control oocytes suggesting that ABA is a substrate for these transporters. NPF4.3 and NPF4.4-expressing oocytes accumulate less ^3^H; this suggests that they behave as ABA efflux transporters. However, this should be confirmed by performing an experiment specifically designed to identify efflux transporter by injecting ABA into the oocytes. The highest accumulation was obtained in oocytes expressing NPF4.5 and NPF4.6 (**Figure 1**).

Since NPF4.5 and 4.6 showed high ABA accumulation, we focused on these two transporters for further characterization.

### Effect of External pH on ABA Accumulation

**Figure 2:**
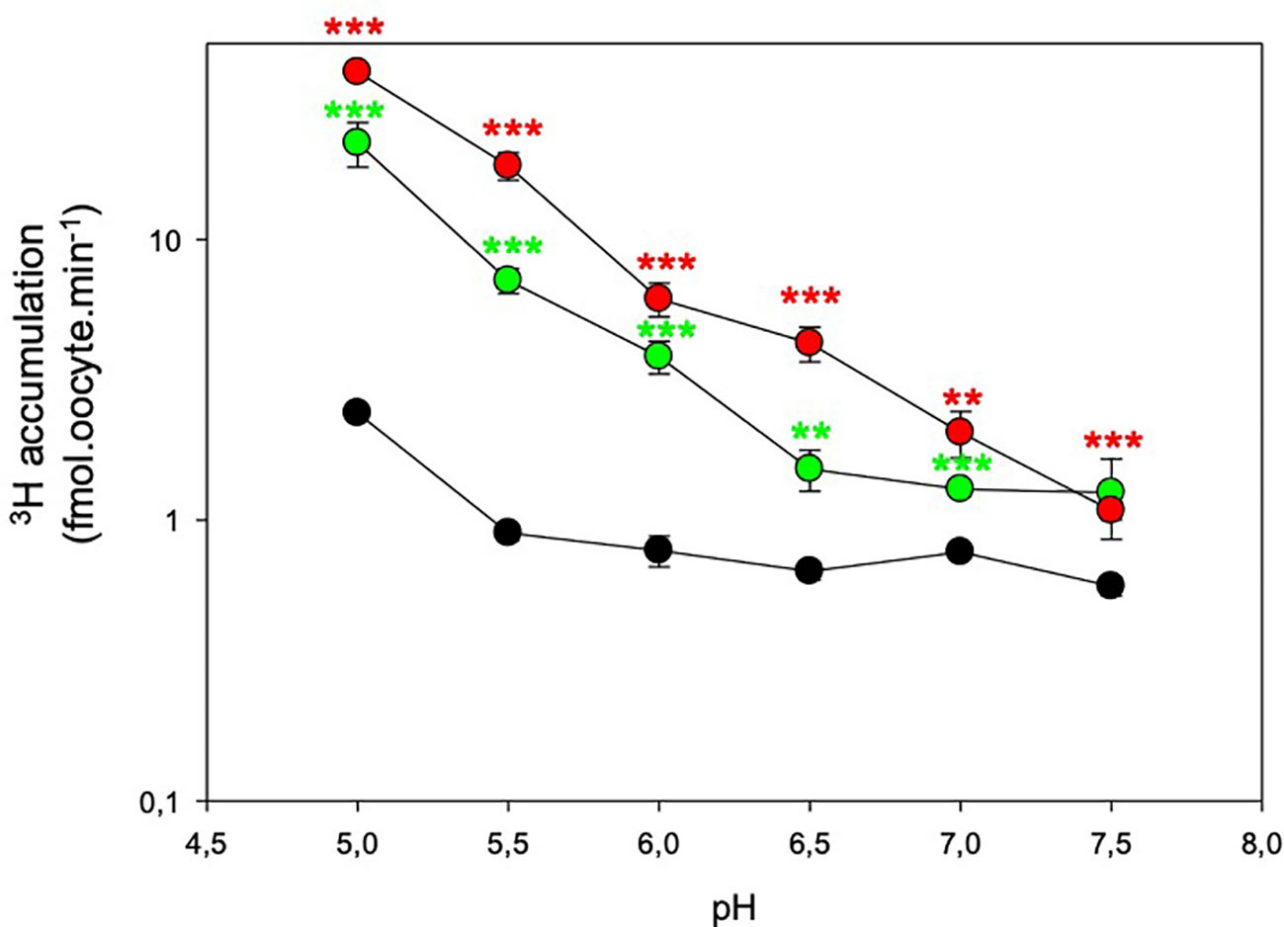
pH-dependent ^3^H accumulation in NPF4.5 and NPF4.6 in Xenopus oocytes. Control (noninjected, black circles), NPF4.5 (green circles) and NPF4.6 (red circles) injected oocytes were bathed in 1 μM ^3^H-ABA (pH = 5.0 to 7.5), and ^3^H accumulation in oocytes was quantified after 20 min. Values are mean +/− SEM (n = 6–20 oocytes, biological replicates). ***P < 0.001, **0.001 < P < 0.005, two-sided *t*-test after comparison with control oocytes.

Most of the NPFs and their animal and bacterial counterparts are proton-coupled transporters. So, we quantified ^3^H accumulation at different external pH, ranging from 5.0 to 7.5 (**Figure 2**). In control oocytes, ^3^H accumulation is not affected in the 5.5–7.5 range and slightly increases at pH 5.0. This is probably due to an increase in the concentration of the protonated form of ABA at acidic pH which increases the membrane diffusion of this form. The external pH sensitivity of NPF4.5 and NPF4.6 is equivalent. ^3^H accumulation is enhanced by acidic pH and NPF-dependent ^3^H accumulation is very low at pH above 7.0.

### ABA Dose Response

**Figure 3:**
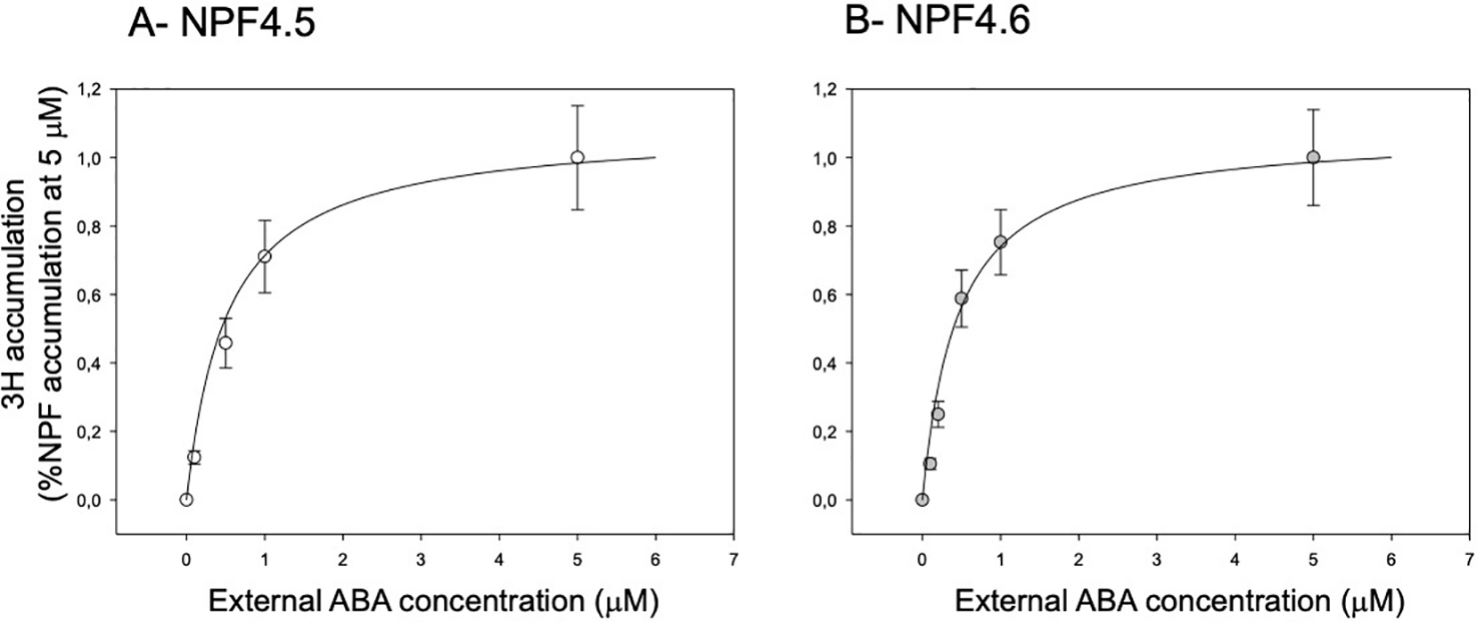
Effect of external ABA concentration on ^3^H accumulation in NPF4.5 and NPF4.6-expressing oocytes. ^3^H-accumulation in NPF4.5 **(A)** and NPF4.6 **(B)** expressing oocytes depending on the wide external ABA concentrations range (0–5 μM ^3^H-ABA). The solid lines are least-squares Michaelian fits. Data are mean +/− SE (n = 6–10 oocytes, biological replicates).

An important property of a transporter is its affinity towards its substrate. We have assessed the apparent affinity (Km) of NPF4.5 and NPF4.6 towards ABA by quantifying ^3^H accumulation into the oocytes at different external ABA concentrations in the 0–5 µM range (**Figure 3**) and at different pH (5.0, 5.5, 6.0, 6.5, 7.0). Data were fitted by a Michaelis–Menten equation: A = (Amax * [ABA])/(Km + [ABA]). This fitting procedure allows determining the apparent affinity of ABA for the transporters (Km). The calculated Km is slightly dependent on the external pH: the Km increases with increase in external pH. At the four tested pH, the Km for both transporters are around 500 nM (**Table 1**).

**Table 1:** Effect of external pH on ABA affinity. ABA accumulations have been determined in different external ABA concentrations (0–5 μM ^3^H-ABA) at 4 different external pH levels: 5.0, 5.5, 6.0, and 6.5.

pH	5.0		5.5		6.0		6.5	
NPF	NPF4.5	NPF4.6	NPF4.5	NPF4.6	NPF4.5	NPF4.6	NPF4.5	NPF4.6
Km (nM)	462	395	485	423	522	452	545	472
± SEM	± 35	± 25	± 47	± 33	± 41	± 25	± 55	± 62

At each pH, data were fitted by a Michaelis–Menten equation: A = (Amax * [ABA])/[Km + [ABA]) to determine the Km. Values are mean +/− SEM (n = 5–12 oocytes, biological replicates).

### Effect of Quinabactin and Pyrabactin on ABA Accumulation

Several ABA-analogs have been identified and characterized (Cao et al., 2017). Within these analogs, pyrabactin and quinabactin induce physiological responses, similar to ABA, through their direct binding to the ABA-receptors from the PYR/PYL/RCAR family. However, nothing is known about their effect on ABA transporters. Direct transport of these ABA-analogs was not possible because there is a no labeled-form of these molecules; so we tested their effect on ABA transport (^3^H accumulation). To test the competition, two concentrations of ABA-analogs were tested at 0.5 and 5 µM in the presence of 1 µM ABA. Neither quinabactin (Okamoto et al., 2013) nor pyrabactin (Park et al., 2009; Kanno et al., 2012) was able to decrease the ^3^H-ABA accumulation into the oocytes, suggesting that they are not transported by, nor bound to NPF4.5 and NPF4.6.

## Discussion

Within the different families of membrane transporters, NPF can transport structurally different substrates (Corratgé-Faillie and Lacombe, 2017). In this family, transporters for different hormones have been identified: auxin, GA, Jasmonate, and ABA. To date, the structure–function relationships are not well defined (Jørgensen et al., 2015; Jørgensen et al., 2017; Longo et al., 2018) and it is not possible to predict the substrate from the sequence. NPF ABA transporters have been characterized in yeast and Sf9 insect cells (Kanno et al., 2012; Kanno et al., 2013; Chiba et al., 2015). These researches identified the NPF4 as a subfamily with several ABA transporters. This work unveils the transport properties of two of these expressed in Xenopus oocytes, AtNPF4.5 and NPF4.6. These data give new insights into the transmembrane transport of ABA influxer. Furthermore, we present our screen of ABA accumulation in oocytes expressing each member of the Arabidopsis NPF4 subfamily. This demonstrates that Xenopus oocytes combined with ^3^H-ABA quantification can be used to study plant ABA transporters.

Our screen confirms that ABA is a substrate for NPF4.5/AIT2, NPF4.6/AIT1/NRT1.2 and NPF4.2/AIT4 (**Figure 1**, Chiba et al., 2015). In our experimental conditions, we were not able to demonstrate an ABA transport activity of NPF4.1/AIT3, further experiments in different conditions should be performed to understand the different results obtained in yeast (Kanno et al., 2012; Kanno et al., 2013; Chiba et al., 2015). The data obtained with NPF4.3 and NPF4.4 should also be studied in more detail. Indeed, in all experiments performed, oocytes expressing these transporters always accumulated less ^3^H (ABA) than control oocytes (**Figure 1**). This is an indication of a putative role in ABA efflux. This could explain the negative results obtained with these transporters expressed in yeast (Kanno et al., 2012; Kanno et al., 2013; Chiba et al., 2015). Finally, NPF4.7 displays ABA transport activity in Xenopus oocytes unlike in yeast (Kanno et al., 2012; Kanno et al., 2013; Chiba et al., 2015). This demonstrates that the use of different heterologous expression systems is a prerequisite to a definitive conclusion about the substrate selectivity of a specific transporter. The functional properties of different plant transporters have been determined in several expression systems and are known to be affected by the expression host [*e.g.* (Dreyer et al., 1999)]. Several explanations have been proposed and it is not possible from our results to discriminate between them: membrane lipid composition, membrane potentials, expression of endogenous regulators (kinases, phosphatases, …), different cytosolic compositions (pH, calcium). In a previous screen (Leran et al., 2015), the nitrate uptake capacity of NPF4.3, 4.3, 4.5, 4.6 was tested, and none of these proteins displayed nitrate transport properties.

We have identified a strong positive effect of external acidification (**Figure 2**) with small effect on the Km (**Table 1**). This could be indicative of an increase in the diffusion of the protonated membrane-permeable form of ABA (ABA-H) because acidification increases its concentration. ABA is a weak acid in equilibrium between the anionic (ABA^−^) form and the protonated (ABA-H) form. The pKa (4.7 for ABA) is the pH at which both forms are at the same concentration (at pH 4.7, 50% of abscisic acid is ABA^−^ and 50% is ABA-H form). At a more acidic pH, ABA-H is the dominant form; whereas at a basic pH [ABA^−^] > [ABA-H]. For example, at pH 7.7, [ABA^−^] = 1000 × [ABA-H]. The protonated form (ABA-H) is uncharged, and therefore, is able to diffuse freely through the membrane lipid bilayer. This phenomenon did not significantly affect ABA accumulation in the control oocyte which is very slightly pH dependent (**Figure 2**). The three other explanations for this are: (i) as most of the NPF characterized so far, NPF4.5 and NPF4.6 are proton coupled transporters, (ii) acidification induces protonation of some amino-acids which induce a modification of the transport properties, and (iii) the transported form of ABA is ABA-H and not the negatively charged ABA^−^. It is not yet possible to determine which one of these explanations is the right one. It could even be a combination of two or three of these hypotheses. Further studies using site-directed mutagenesis will give the opportunity to test these hypotheses.

The dose response curve of ABA transport activity *versus* the external ABA concentration follows a Michaelis–Menten behavior (**Figure 3**). Fitting the data allows determining the affinity: the Km is *ca.* 500 nM for both transporters and is only slightly modified by external pH (**Table 1**). This is 10-fold lower than what has been previously determined by NPF4.6-expression in yeast [5 µM, (Kanno et al., 2012)]. These experiments in yeast have been performed at pH 7.5, whereas our experiments were done at pH 6.0. We cannot test this in oocytes because, at pH 7.5, there is no NPF-dependent ^3^H accumulation. But the small change in Km in the 5.0–6.5 range (**Table 1**) does not support the fact that the difference in Km observed in yeast and in oocyte is explained by a different external pH.

**Figure 4:**
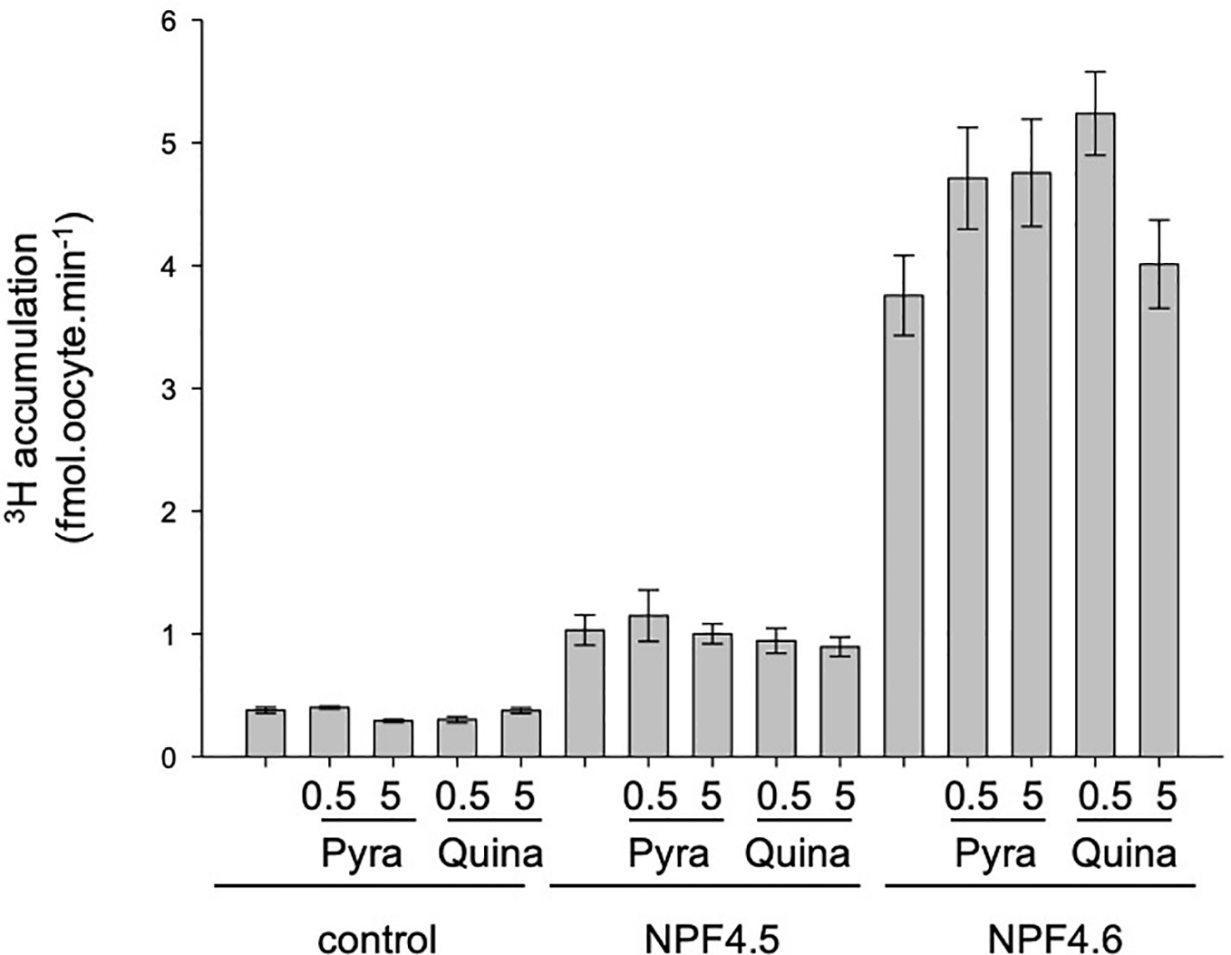
Effect of pyrabactin and quinabactin on ^3^H accumulation in NPF4.5 and NPF4.6 in Xenopus oocytes. Control (noninjected), NPF4.5 and NPF4.6 injected oocytes were bathed in 1 μM ^3^H-ABA (pH = 6.0), and ^3^H accumulation in oocytes was quantified after 20 min in the presence or in the absence of 0.5 or 5 μM of pyrabactin or quinabactin. Values are mean +/− SEM (n = 9–11 oocytes, biological replicates). The pyrabactin and quinabactin treatment has no significant effect on ^3^H accumulation (two-sided *t*-test).

The selectivity and affinity of ABA receptors have been studied, and several ABA analogs with higher affinity for the receptors have been identified, as pyrabactin and quinabactin (**Figure 4**) (Park et al., 2009; Okamoto et al., 2013). The effect of these molecules on ABA transport has been tested. They have no effect on ^3^H accumulation, suggesting that (i) they are not competitive inhibitors of NPF-dependent ABA transport and (ii) they are not transported. However, the development of labeled forms of these molecules is necessary to confirm the absence of transport.

Xenopus oocytes have been used to characterize plant transporters from different transporter families (Larsen et al., 2017). The possibility to use this convenient system for most plant hormones is now established (Wulff et al., 2019), and specific drawbacks have been recently identified (Wulff et al., 2019). This system can be used to characterize ABA transport from the NPF family and will be used to perform a structure–function analysis to identify the amino-acids involved in the ABA selectivity of these transporters. It will be also interesting to study the properties of the ABA transporter from the ABCG family expressed in Xenopus oocytes. Some more data should also be obtained *in planta* to have a better understanding of the transport properties in different tissues (Boursiac et al., 2013). 

## Data Availability Statement

The datasets generated for this study are available on request to the corresponding author.

## Author Contributions

SL, MN, CC-F, YB, CB, and BL performed the research and analyzed the data. BL conceived the work and wrote the manuscript.

## Funding

This work was supported by the Institut National de la Recherche Agronomique (CJS PhD Fellowship to SL & Projet Département BAP, BAP2013-33-NITSE to BL), Agence Nationale de la Recherche (ANR-11-JSV6-002-01-NUTSE and ANR-14-CE34-0007-01-HONIT to BL) and the Région Languedoc-Roussillon (Chercheur d'Avenir to BL).

## Conflict of Interest

The authors declare that the research was conducted in the absence of any commercial or financial relationships that could be construed as a potential conflict of interest.
